# Colonization characteristics of fungi in *Polygonum hydropipe* L. and *Polygonum lapathifolium* L. and its effect on the content of active ingredients

**DOI:** 10.3389/fpls.2022.984483

**Published:** 2022-09-28

**Authors:** Xiaorui Zhang, Hongyang Lv, Maoying Tian, Zhaowei Dong, Qinwen Fu, Jilin Sun, Qinwan Huang, Jin Wang

**Affiliations:** ^1^State Key Laboratory of Characteristic Chinese Medicine Resources in Southwest China, Chengdu University of Traditional Chinese Medicine, Chengdu, China; ^2^Pharmacy College, Chengdu University of Traditional Chinese Medicine, Chengdu, China; ^3^Sichuan Fuzheng Pharmaceutical Co., Ltd., Chengdu, China

**Keywords:** *Polygonum hydropiper* L., endophytic fungi, community assembly process, flavonoids, core community

## Abstract

*Polygonum hydropiper*, is a plant of the Persicaria genus, which is commonly used to treat various diseases, including gastrointestinal disorders, neurological disorders, inflammation, and diarrhea. However, because of different local standards of *P. hydropiper*, people often confuse it with *Polygonum lapathifolium* L. and other closely related plants. This poses a serious threat to the safety and efficacy of the clinical use of *P. hydropiper*. This study aims to determine the six active ingredients of *P. hydropiper* and *P. lapathifolium*. Then the endophytic fungi and rhizosphere soil of the two species were sequenced by Illumina Miseq PE300. The results show significant differences between the community composition of the leaves, stems, and roots of the *P. hydropiper* and the *P. lapathifolium* in the same soil environment. Of the six secondary metabolites detected, five had significant differences between *P. hydropiper* and *P. lapathifolium*. Then, we evaluated the composition of the significantly different communities between *P. hydropiper* and *P. lapathifolium*. In the *P. hydropiper*, the relative abundance of differential communities in the leaves was highest, of which *Cercospora* dominated the differential communities in the leaves and stem; in the *P. lapathifolium*, the relative abundance of differential community in the stem was highest, and *Cladosporium* dominated the differential communities in the three compartments. By constructing the interaction network of *P. hydropiper* and *P. lapathifolium* and analyzing the network nodes, we found that the core community in *P. hydropiper* accounted for 87.59% of the total community, dominated by *Cercospora*; the core community of *P. lapathifolium* accounted for 19.81% of the total community, dominated by *Sarocladium*. Of these core communities, 23 were significantly associated with active ingredient content. Therefore, we believe that the community from *Cercospora* significantly interferes with recruiting fungal communities in *P. hydropiper* and affects the accumulation of secondary metabolites in the host plant. These results provide an essential foundation for the large-scale production of *P. hydropiper*. They indicate that by colonizing specific fungal communities, secondary metabolic characteristics of host plants can be helped to be shaped, which is an essential means for developing new medicinal plants.

## Introduction

*Polygonum hydropiper* is a plant of the *Persicaria* genus in the family *Polygonaceae*. The whole plant of *P. hydropiper* is commonly used to treat various diseases, including gastrointestinal disorders, neurological disorders, inflammation, and diarrhea (Xiang and Ming, 2020). Due to the low requirements for growth conditions, it has abundant wild plant resources what is more, it is widely distributed in China in Sichuan, Guangdong, Guangxi, and other places (Hong and Hanshen, 2013). Many studies have shown that *P. hydropiper* is rich in various chemical components, including flavonoids, terpenes, and organic acids, with antimicrobial, antioxidant, antiviral, insecticidal, and other biological activities (Rahman et al., 2002; Ayaz et al., 2014; Sharif et al., 2014; Shahed-Al-Mahmud and Lina, 2017). Due to different local standards, the large-scale commercial cultivation of *P. hydropiper* has not yet been achieved. People are often confused by plants, such as *Polygonum lapathifolium* and *P. orientale* (Hong et al., 2019). This poses a serious threat to the safety and efficacy of the clinical use of *P. hydropiper*. Therefore, clarifying the differences between *P. hydropiper* and other obfuscated species is crucial to developing and improving the production of *P. hydropiper*.

Our preliminary research found that as one of the drugs for the treatment of enteritis, the role of flavonoids in it cannot be ignored (Zhang et al., 2021). Quercetin, kaempferol, isorhamnetin, hyperoside, catechins, and chlorogenic acid are the main active ingredients of *P. hydropiper* in treating enteritis (Yue, 2005; Wei et al., 2021). It is worth noting that Tian et al. (2014) found the potential of flavonoids to act as signaling molecules between endophytic fungi and plants. Meanwhile, Tang et al. (2020) found that the endophyte isolated from Conyza blinii H. Lév could produce flavonoids with high yield and excellent biological activity. Endophytic fungi are present in all plants and, together with host plants, determine the production of secondary metabolites (Waqas et al., 2012; Adeleke and Babalola, 2021). With the development of next-generation sequencing, there is increasing evidence that the compositional pattern of endophytic fungi is related to the production of specific secondary metabolites (Lunardelli Negreiros de Carvalho et al., 2016; Dang et al., 2021; Ribeiro et al., 2021). In some crops and medicinal plants, it is also recognized that the specific community composition pattern can reflect the quality and yield of the host plant (Song et al., 2010; Vergara et al., 2018; Cao et al., 2021; Martins et al., 2021).

There are abundant researches of chemical composition and pharmacological effect research of *P. hydropiper* (Hong and Hanshen, 2013). However, its endophytic fungi research is still blank. Thus, this experiment uses Illumina Miseq PE300 sequencing technology to sequence the endophytic fungi of *P. hydropiper* and *P. lapathifolium*. At the same time, we measured the content of 6 flavonoids of two species to reveal the relationship between the endophytic fungal community composition and the accumulation of active ingredients. Furthermore, determining the composition of the core microbiota related to active ingredients to improve the production of *P. hydropiper* provides key fundamental data.

## Methods

### Sampling

In July 2021, the whole plant and rhizosphere soil of *P. hydropiper* (ZP) and *P. lapathifolium* (WP) were sampled from Huoba Village in Jianyang City, China. At each sample point, four repeats of the whole plant and three repeats of the rhizosphere soil were collected. The rhizosphere soil was collected by the jitter root method. The plant residue and gravel were selected and discarded, and the samples were collected by the quartering method and loaded into sterile centrifuge tubes. To ensure the representativeness of the samples and avoid edge effects, we set the sample point away from the field ridge at least 25 m and each sample point interval at least 2 m. Samples are frozen with liquid nitrogen after on-site sampling and quickly transported back to the laboratory on dry ice for preservation at −80°C (Table 1).

**Table 1:** Samples information.

**Samples**	**Type**	**Date**	**Site**
ZY-1	Leaf	2020/7/6	Huoba Village in Jianyang City, China
ZY-2	Leaf	2020/7/6	Huoba Village in Jianyang City, China
ZY-3	Leaf	2020/7/6	Huoba Village in Jianyang City, China
ZY-4	Leaf	2020/7/6	Huoba Village in Jianyang City, China
ZJ-1	Stem	2020/7/6	Huoba Village in Jianyang City, China
ZJ-2	Stem	2020/7/6	Huoba Village in Jianyang City, China
ZJ-3	Stem	2020/7/6	Huoba Village in Jianyang City, China
ZJ-4	Stem	2020/7/6	Huoba Village in Jianyang City, China
ZG-1	Root	2020/7/6	Huoba Village in Jianyang City, China
ZG-2	Root	2020/7/6	Huoba Village in Jianyang City, China
ZG-3	Root	2020/7/6	Huoba Village in Jianyang City, China
ZG-4	Root	2020/7/6	Huoba Village in Jianyang City, China
WY-1	Leaf	2020/7/6	Huoba Village in Jianyang City, China
WY-2	Leaf	2020/7/6	Huoba Village in Jianyang City, China
WY-3	Leaf	2020/7/6	Huoba Village in Jianyang City, China
WY-4	Leaf	2020/7/6	Huoba Village in Jianyang City, China
WJ-1	Stem	2020/7/6	Huoba Village in Jianyang City, China
WJ-2	Stem	2020/7/6	Huoba Village in Jianyang City, China
WJ-3	Stem	2020/7/6	Huoba Village in Jianyang City, China
WJ-4	Stem	2020/7/6	Huoba Village in Jianyang City, China
WG-1	Root	2020/7/6	Huoba Village in Jianyang City, China
WG-2	Root	2020/7/6	Huoba Village in Jianyang City, China
WG-3	Root	2020/7/6	Huoba Village in Jianyang City, China
WG-4	Root	2020/7/6	Huoba Village in Jianyang City, China
ZT-1	Rhizosphere soil	2020/7/6	Huoba Village in Jianyang City, China
ZT-2	Rhizosphere soil	2020/7/6	Huoba Village in Jianyang City, China
ZT-3	Rhizosphere soil	2020/7/6	Huoba Village in Jianyang City, China
WT-1	Rhizosphere soil	2020/7/6	Huoba Village in Jianyang City, China
WT-2	Rhizosphere soil	2020/7/6	Huoba Village in Jianyang City, China
WT-3	Rhizosphere soil	2020/7/6	Huoba Village in Jianyang City, China

### Determination of active ingredients content

#### Chemicals and reagents

Six commercial standards of HPLC grade, including catechins, chlorogenic acid, hyperoside, quercetin, kaempferol, and isorhamnetin, were purchased from Chengdu Pufei De Biotech Co., Ltd. (China). Other HPLC grade reagents used were acetonitrile and methanol from Thermo Fisher Scientific Co., Ltd. (USA). The Watsons distilled water was applied to prepare for samples and the mobile phase. All other reagents were just met for an analytical grade.

#### Sample preparation

We precisely weighed 0.1 g of PL sample, added it to 15 mL of 60% ethanol in an Erlenmeyer flask, and dissolved it by sonication at 50°C for 30 min. Then filtering the sample, and evaporated the filtrate. The methanol was added to the residue until the volume to 5 mL, and the solution was filtered with a microporous filter membrane of 0.22 μm.

#### UPLC-QQQ-MS/MS analysis

Ultra performance liquid chromatograph LC-20A and triple quadrupole mass spectrometer LCMS-8045 were purchased from Shimadzu Co., Ltd. (Japan). A Shim-pack Velox C18 column (2.1 × 1,000 mm, 2.7 μm) was employed at a column temperature of 30°C. The mobile phase consisted of 0.3% carboxylic acid in water (A) and methanol (B), and the flow rate was 0.2 mL/min. The gradient elution parameters were set as follows: 0–6 min, 15% B; 6–15 min, 15–45% B; 15–20 min, 45%– 47% B; 20–22 min, 47%−50% B; 22–25 min, 50%−15% B. The injection volume was 2 μL.

Ionization method: Electrospray ion source (ESI); Multi-reaction monitoring mode (MRM); Curtain Gas (CUR) flow rate: 40 L·min^−1^; Atomized Gas (GS1) flow rate: 55 L·min^−1^; Auxiliary gas (GS2) flow rate: 55 L·min^−1^; ionization temperature (TEM): 550°C; Spray voltage (IS): 4,500 V in positive ion mode, −4,500 V in negative ion mode. The optimized MS/MS parameters of ZP are shown in Table 1.

#### Method validation

The six reference standards were weighed accurately and dissolved with methanol comparable to sample extracts. Calibration curves were constructed by measuring the signal intensity (peak area) of MRM transitions for at least six appropriate concentrations of each compound. Intra-day and inter-day precisions were evaluated by calculating the relative standard deviations (RSDs) of retention time and signal intensity during a single day and on three successive days, respectively. Repeatability was evaluated by calculating the RSDs of retention time and signal intensity of six tested solutions made from the same sample on a single day. Stability was evaluated by calculating the RSDs of retention time and signal intensity of the same tested solution during a single day at 0, 2, 4, 8, 12, and 24 h. Recovery experiments were done by spiking authentic standards into samples directly.

### DNA extraction and library construction

Plants and soil samples were finely ground to powder in liquid nitrogen using a tissue grinder separately, and 0.5 g was taken for DNA extraction using the FastDNA^®^SPIN Kit (MP Biomedicals, US). Three repeats per sample were required. The DNA bands, concentration, and purity of the extract were detected using a 1% agarose gel electrophoresis and an accounting analyzer, and samples with a concentration of ≥20 ng/μL were selected and sent to Shanghai Majorbio Bio-pharm Technology Co., Ltd for Polymerase Chain Reaction (PCR) amplification, DNA sequencing, and library construction. Fungal ITSregion was amplified using the forward primer ITS1F (CTTGGTCATTTAGAGGAAGTAA) and reverse primer ITS2R (GCTGCGTCTTCATCGATGCGC). The PCR reaction mixture, including 2 μL 10 × buffer, 2 μL 2.5 mM dNTPs, 0.8 μL each primer (5 μM), 0.2 μL rTaq polymerase, 0.2 μL BSA, 10 ng of template DNA, and ddH_2_O to a final volume of 20 μL. PCR amplification cycling conditions were as follows: initial denaturation at 95°C for 3 min, followed by 35 cycles of denaturing at 95°C for 30 s, annealing at 55°C for 30 s and extension at 72°C for 45 s, and single extension at 72°C for 10 min, and end at 4°C. All samples were amplified in triplicate. The PCR product was extracted from a 2% agarose gel and purified using the AxyPrep DNA Gel Extraction Kit (Axygen Biosciences, Union City, CA, USA) according to the manufacturer's instructions and quantified using Quantus™ Fluorometer (Promega, USA). Purified amplicons were pooled in equimolar amounts and paired-end sequenced on an Illumina MiSeq PE300 platform (Illumina, San Diego, USA) according to the standard protocols by Majorbio Bio-Pharm Technology Co. Ltd. (Shanghai, China). The data presented in the study are deposited in the NCBI Sequence Read Archive (SRA) database repository, accession number SRR20897189-SRR20897218.

### Data processing

Clean reads were obtained by filtering the raw sequences using a microbial ecological quantitative analysis pipeline (QIIME, version 1.9.1, USAU). Low-quality sequences (such as uncertain nucleotide sequences, three nucleotides with a *Q*-value of < 20, and unmatched barcode sequences) were removed. The QIIME v1.9.0 was used for quality control to obtain valid data, and the Uchime algorithm and gold database were used to remove delusion. These sequences were grouped into operational taxonomic units (OTUs) based on 97% sequence identity using UPARSE (V7.0.1090). Each row was annotated by comparing the Ribosomal Database Project (RDP) classifier (V2.11) against the unite8.0 database using a comparison threshold of 70%. Resampling was carried out with the smallest amount of data in the sample as the standard to make the uniform treatment for each sample. Mothur (version 1.30.2) 1 was used for diversity analysis. R 3.6.0 was used to perform various data conversions. Fungi Functional Guild (FUNGuild) was used for function prediction.

### Statistical analysis

All statistical analyses were performed in R (v4.0.3) (Team, 2020). Hellinger transformation was first used to convert microbiota data. The Alpha diversity and Principal Component Analysis (PCA) analysis were generated by the vegan package in R (Oksanen et al., 2013) and plotted by ggplot2 (Wickham, 2011). The ternary diagram was drawn using the ggtern in R (Hamilton and Ferry, 2018). The significant difference between the functional composition of ZP and WP were detected by the Wilcoxon test. The significant different taxa between ZP and WP were detected by the MetagenomeSeq package (Wickham, 2011). Then, we performed the Pearson correlation analysis between the core community and the ingredients. Draw the heatmap of the correlation analysis using the Pheatmap package (Kolde and Kolde, 2015), and remove the community not significantly.

### Interaction network analysis

We first use the iGraph package (Csardi and Nepusz, 2006) to analyze the network structure of the endophytic fungi community of *P. hydropiper* and *P. lapathifolium*. Then we use the weighted gene co-expression network analysis (WGCNA) package (Langfelder and Horvath, 2008) to calculate the correlation and *P*-values. And use the false discovery rates (FDR) to correct the *P*-values. The interaction network was generated by retaining the edges of *R* ≥ ±0.4 and *P* ≤ 0.05. Then we import the interaction network into Cytoscape (Shannon et al., 2003) to analyze the module. We choose the fast greedy algorithm to calculate the module, and use the GuImerà Amaral NeTwork (GIANT) package (Cumbo et al., 2014) to analyze the module's connectivity and the connectivity between modules. Finally, we use the ggplot2 package to plot the results.

## Results

### Determination of active ingredient content

#### Method validation

**Figure 1:**
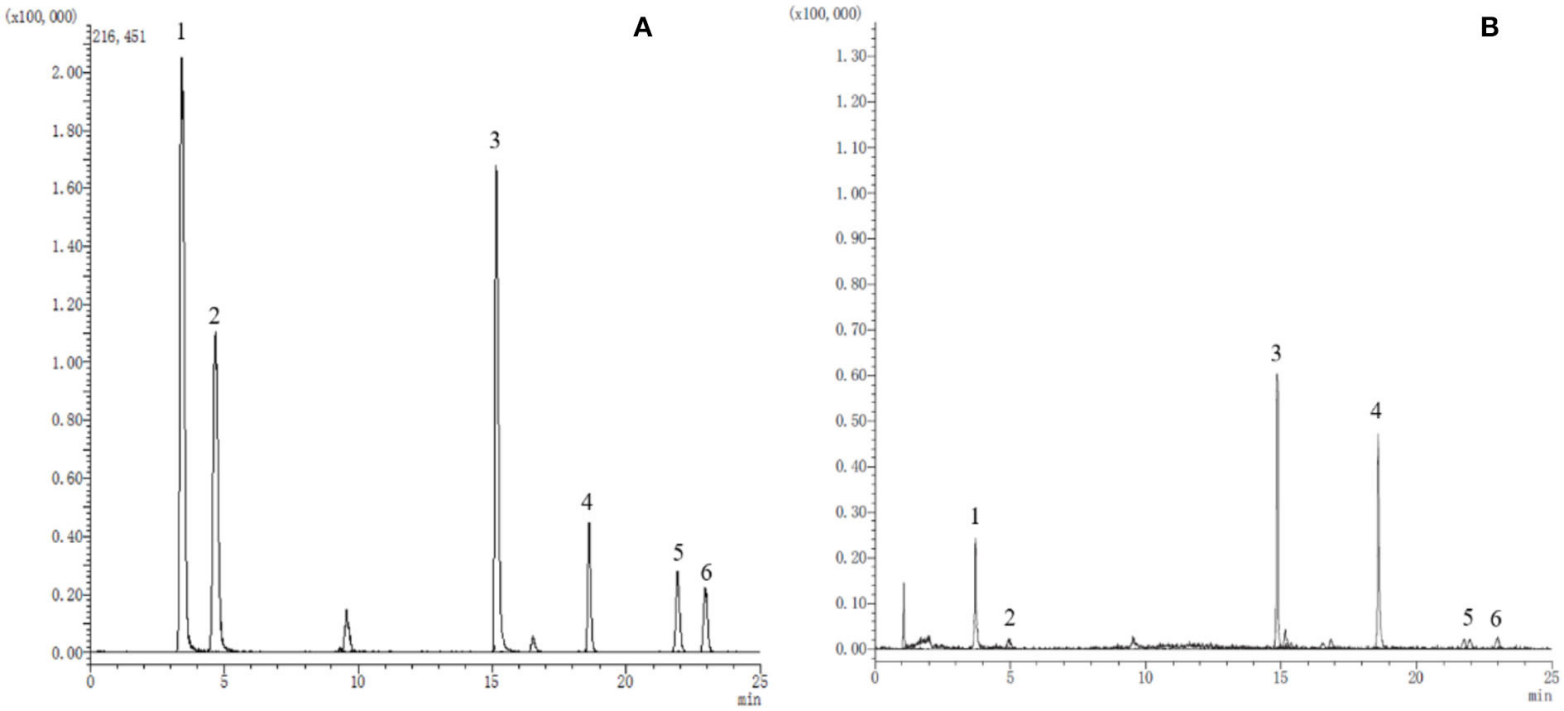
The extracted ion chromatograms (EICs) of reference substances **(A)** and sample **(B)**. 1. Catechin; 2. Chlorogenic acid; 3. Hyperoside; 4. Quercetin; 5. Kaempferol; 6. Isorhamnetin.

A preliminary optimization of the UPLC method (flow rate, gradient, injection volume, etc.) successfully achieved a well-separated peak for every standard compound, as shown in Figure 1. Moreover, the performance validation of the method was evaluated, including linearity, precision, reproducibility, stability, and recovery (Table 2). The linearity of the method was measured by analyzing standards over a linear range suitable for quantifying the corresponding analytes. Good linearity between concentration and signal intensity was obtained, and correlation coefficients of all compounds were calculated to be more than 0.999. Additionally, good reproducibility, stability, and precision were revealed in the results.

**Table 2:** Liner equations, precision, repeatability, stability, and average recovery rates of quantification of six components.

**Compound**	**Liner equations**	***R***	**Precision (RSD/%)**		**Repeatability RSD/%**	**Stability RSD/%**	**Recovery**	
			**Intra-day (n = 6)**	**Inter-day (n = 9)**			**Mean/%**	**RSD/%**
Catechins	Y = 55091.2X + 1252.94	0.9991	2.5	2.59	2.96	1.86	92.02	2.14
Chlorogenic acid	Y = 17261.2X + 22440.6	0.9996	2.73	2.42	2.58	2.58	102.45	2.75
Hyperoside	Y = 2982.38X + 29865.3	0.9992	2.87	2.57	0.60	0.11	100.62	2.41
Quercetin	Y = 4467.8X + 11024.8	0.9995	2.72	2.66	2.17	1.53	102.47	2.65
Kaempferol	Y = 12145.9X + 18648.7	0.9993	2.15	2.20	2.04	1.35	97.37	2.96
Isorhamnetin	Y = 28372.1X + 13477.73	0.9993	1.43	2.76	1.88	2.02	97.52	2.06

The above results suggest that the established method is sensitive, rapid, and reliable in identifying and quantifying phenolic compounds. The MRM mode of the UPLC-QQQ-MS/MS system is an effective and efficient tool for natural product analysis in complex matrices.

#### Sample content determination

The content determination results of catechins, chlorogenic acid, hyperoside, quercetin, kaempferol, and isorhamnetin are shown in four batches of ZP and four batches of WP in Table 3. In addition, we used student's *t*-test to detect the significant difference between the content of the active compounds in the two groups of ZP and WP, and the results are shown in Table 4.

**Table 3:** The content of the six compounds in ZP and WP.

	**Catechins (μg·g−1)**	**Chlorogenic acid (μg·g−1)**	**Hyperoside (μg·g−1)**	**Quercetin (μg·g−1)**	**Kaempferol (μg·g−1)**	**Isorhamnetin (μg·g−1)**
Z1	17.3281	3.5837	501.1931	366.8620	30.9806	24.3484
Z2	17.5247	3.7231	507.4762	389.4847	30.7550	25.9680
Z3	17.4783	2.7570	508.8720	361.1795	22.5175	25.7283
Z4	16.7874	2.5801	519.0559	334.8053	18.7093	24.1923
W1	29.2366	1.1047	225.8444	184.4371	15.9720	13.5882
W2	33.4035	1.1563	215.0943	190.2311	14.0855	10.4824
W3	36.5700	1.3370	234.7462	197.7751	23.6914	13.6166
W4	37.6574	1.2769	204.4158	184.9176	19.7679	7.8025

**Table 4:** Student's *t*-test of component content between ZP and WP.

**Compounds**	**ZP**		**WP**		***P*-value**
	**Mean**	**SD**	**Mean**	**SD**	
Catechins	17.2796	0.3387	34.2169	3.7789	0.0028
Chlorogenic acid	3.1610	0.5760	1.2187	0.1069	0.0057
Hyperoside	509.1493	7.4009	220.0252	13.1471	0.0000
Quercetin	363.0829	22.4691	189.3402	6.2059	0.0003
Kaempferol	25.7406	6.1218	18.3792	4.2575	0.1016
Isorhamnetin	25.0593	0.9184	11.3724	2.7978	0.0012

For ZP, the catechins content was between 16.7874 and 17.5247 μg g^−1^, the chlorogenic acid content was between 2.5801 and 3.7231 μg g^−1^, the content of hyperoside was between 501.1931 and 519.0559 μg g^−1^, the quercetin content was between 334.8053 and 389.4847 μg g^−1^, and the content of kaempferol was between 18.7093 and 30.9806 μg g^−1^. The content of isorhamnetin was between 24.1923 and 25.9680 μg g^−1^; for WP, the content of catechins was between 29.2366 and 37.6574 μg g^−1^; the chlorogenic acid content was between 1.1047 and 1.3370 μg g^−1^; the content of hyperoside was between 204.4158 and 234.7462 μg g^−1^; the quercetin content as between 184.4371 and 197.7751 μg g^−1^; the content of kaempferol was between 14.0855 and 23.6914 μg g^−1^, and the content of isorhamnetin was between 7.8025 and 13.6166 μg·g^−1^.

Student's *t*-test results (Table 4) showed that the contents of chlorogenic acid, hyperoside, quercetin, and isorhamnetin in ZP were significantly higher than those in WP, and the catechins in WP were considerably higher than those in ZP, while there was no significant difference in kaempferol between the two species.

### Statistics of ITS amplicon sequencing

**Figure 2:**
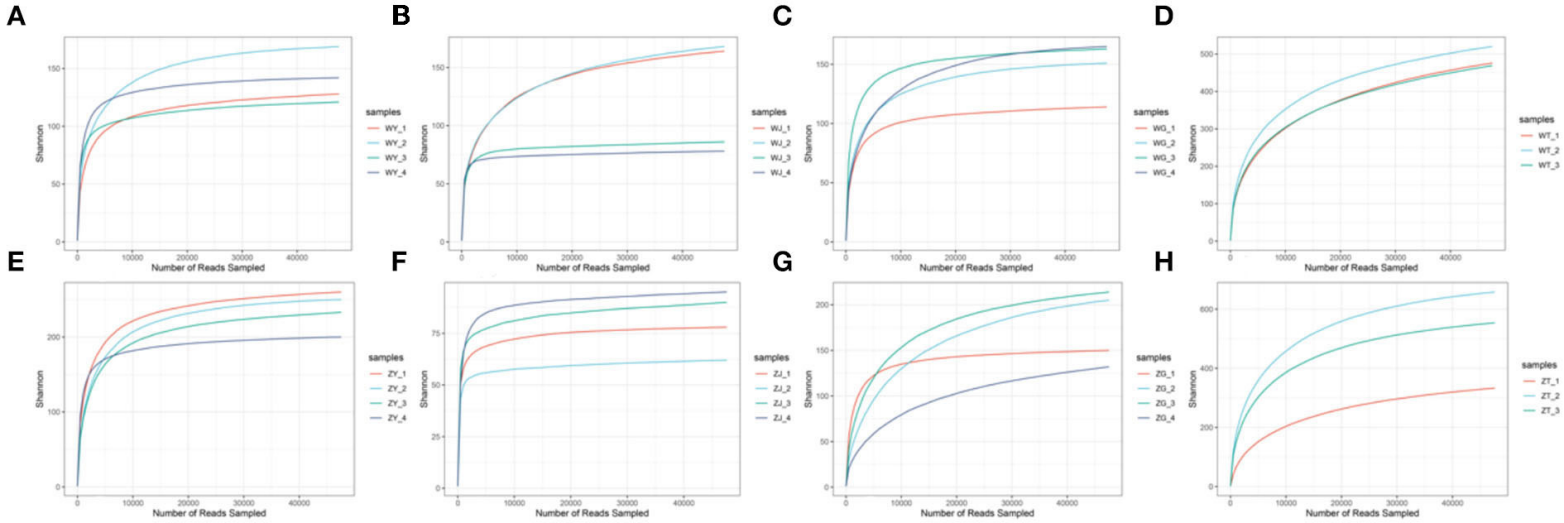
The rarefaction curve [**(A)** the leaf of WP; **(B)** the stem of WP; **(C)** the root of WP; **(D)** the rhizosphere soil of WP; **(E)** the leaf of ZP; **(F)** The stem of ZP; **(G)** the root of ZP; **(H)** the rhizosphere soil of ZP].

We sequenced 30 plant and soil samples, and a total of 2,321,991 reads were obtained, with a total base number of 696,597,300 bp. After filtering, the clean reads were 2,321,991, the total number of bases was 540,788,432 bp, and the average length was 232 bp. The number of sequences assigned to each sample was 47,618–169,166, with a median of 75,882 (Table 5). Based on the minimum sample sequence size, 1,642 OTUs were obtained at a similarity of 97%. The rarefaction curve showed sufficient sequencing depth (Figure 2), and the Good's coverage also shows good taxa coverage for each sample (Table 5).

**Table 5:** Sequencing statistics.

	**Compartment**	**No**.	**Sequence number**	**Sequence base number**	**Average length**	**Min length**	**Max Length**	**Coverage**
ZP	Leaf	1	81671	18659755	228.47	150	514	99.98%
	Leaf	2	75151	17275810	229.88	149	514	99.96%
	Leaf	3	67298	15158745	225.25	144	473	99.98%
	Leaf	4	80201	18570459	231.55	152	524	99.97%
	Rhizosphere soil	1	52439	12505425	238.48	165	517	99.84%
	Rhizosphere soil	2	60256	13933186	231.23	143	518	99.83%
	Rhizosphere soil	3	54748	13127476	239.78	140	522	99.84%
	Stem	1	47618	11099330	233.09	164	515	99.99%
	Stem	2	69632	16378460	235.21	173	505	99.99%
	Stem	3	63497	14935152	235.21	171	523	99.99%
	Stem	4	57766	13408123	232.11	172	423	99.99%
	Root	1	64740	16161710	249.64	173	494	99.93%
	Root	2	57239	16812244	293.72	146	395	99.94%
	Root	3	76613	17766891	231.90	145	349	99.93%
	Root	4	169166	42516049	251.32	150	438	99.99%
WP	Leaf	1	69738	16457499	235.99	141	321	99.99%
	Leaf	2	91724	20805391	226.83	169	504	99.99%
	Leaf	3	80418	18451726	229.45	157	321	99.98%
	Leaf	4	88068	20608782	234.01	181	393	99.98%
	Rhizosphere soil	1	72261	16224948	224.53	142	525	99.77%
	Rhizosphere soil	2	66798	15601070	233.56	141	433	99.79%
	Rhizosphere soil	3	63371	14296112	225.59	141	528	99.76%
	Stem	1	94776	20978893	221.35	154	531	99.99%
	Stem	2	96825	21836843	225.53	169	526	99.99%
	Stem	3	82421	18990185	230.40	187	528	99.95%
	Stem	4	76992	17647509	229.21	191	522	99.96%
	Root	1	85574	19244983	224.89	155	498	99.98%
	Root	2	76856	17077185	222.20	177	366	99.98%
	Root	3	100743	22772037	226.04	168	529	99.99%
	Root	4	97391	21486454	220.62	169	507	99.99%

### Analysis of the diversity and composition of the fungal communities

**Figure 3:**
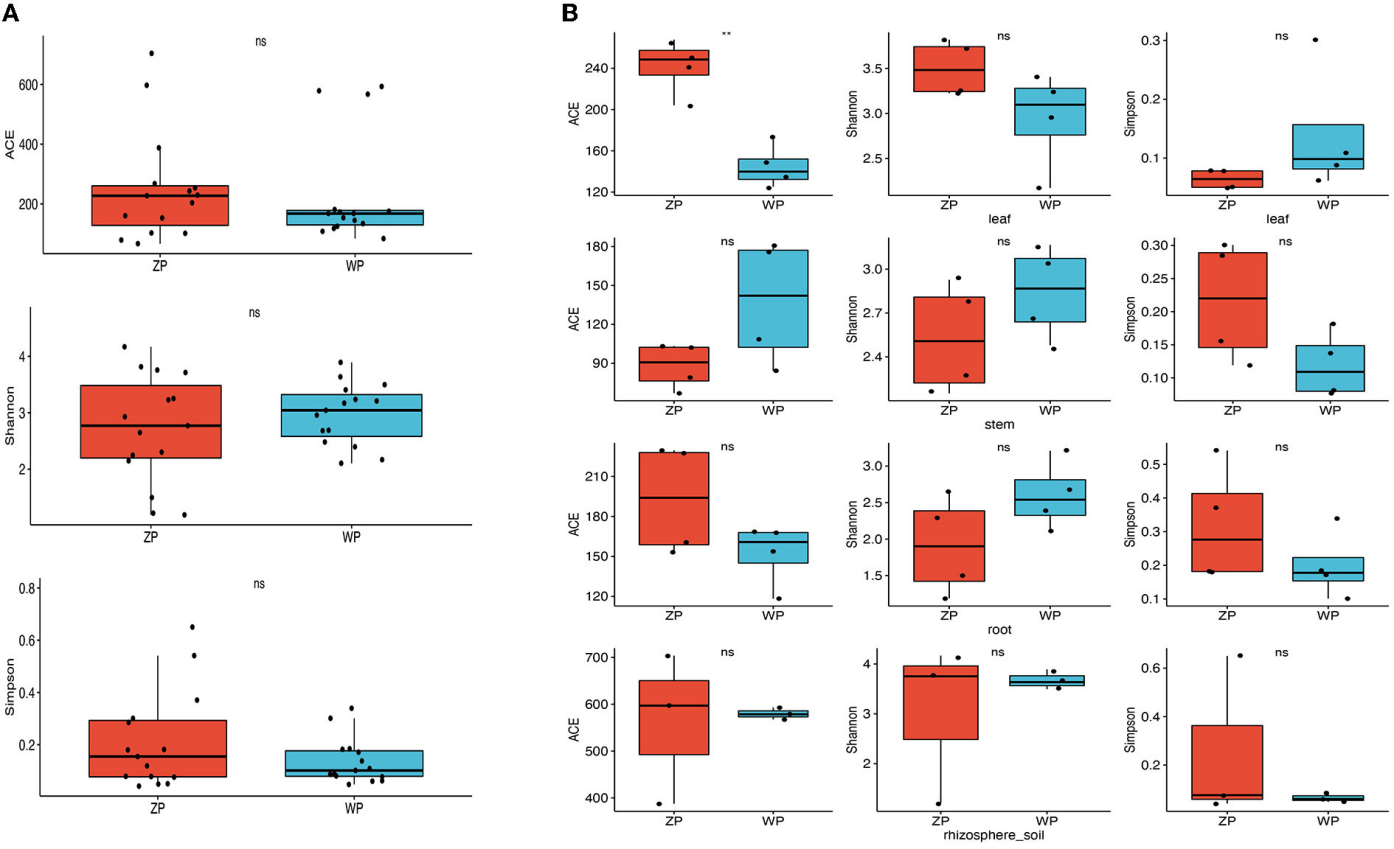
Alpha diversity of fungal communities; **(A)** Difference analysis between whole plant of two species; **(B)** Difference analysis between three compartments of two species. *Means P-value < 0.05, **means P-value < = 0.01, ***means p-value < = 0.001, ****means P-value < = 0.0001, ns mean P-value > 0.05.

To compare the differences in fungal communities of two plants from a macroscopic perspective. First, we compared the difference in the alpha diversity index of all samples of the two species. As can be seen from Figure 3A, there are no significant differences in the ACE, Shannon, and Simpson indices between the two species. Then, the difference in the alpha diversity of the same compartment of the two species was compared, and it can be seen from Figure 3B that there is a significant difference in the ACE index between ZP and WP in the leaves.

**Figure 4:**
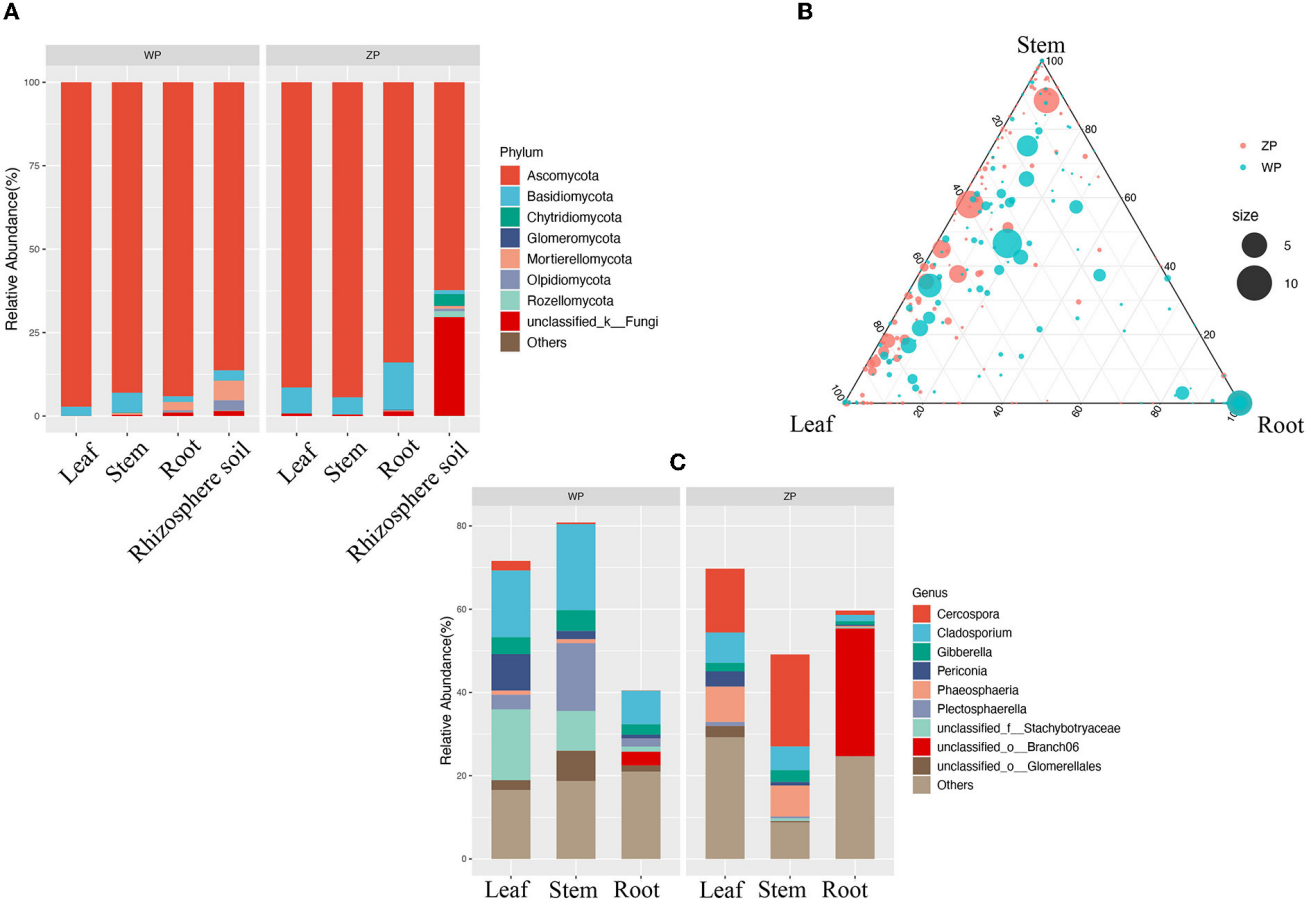
**(A)** The community composition of ZP and WP at the phylum level; **(B)** The ternary phase diagram of community in ZP and WP; **(C)** The significant differential communities between ZP and WP.

**Figure 5:**
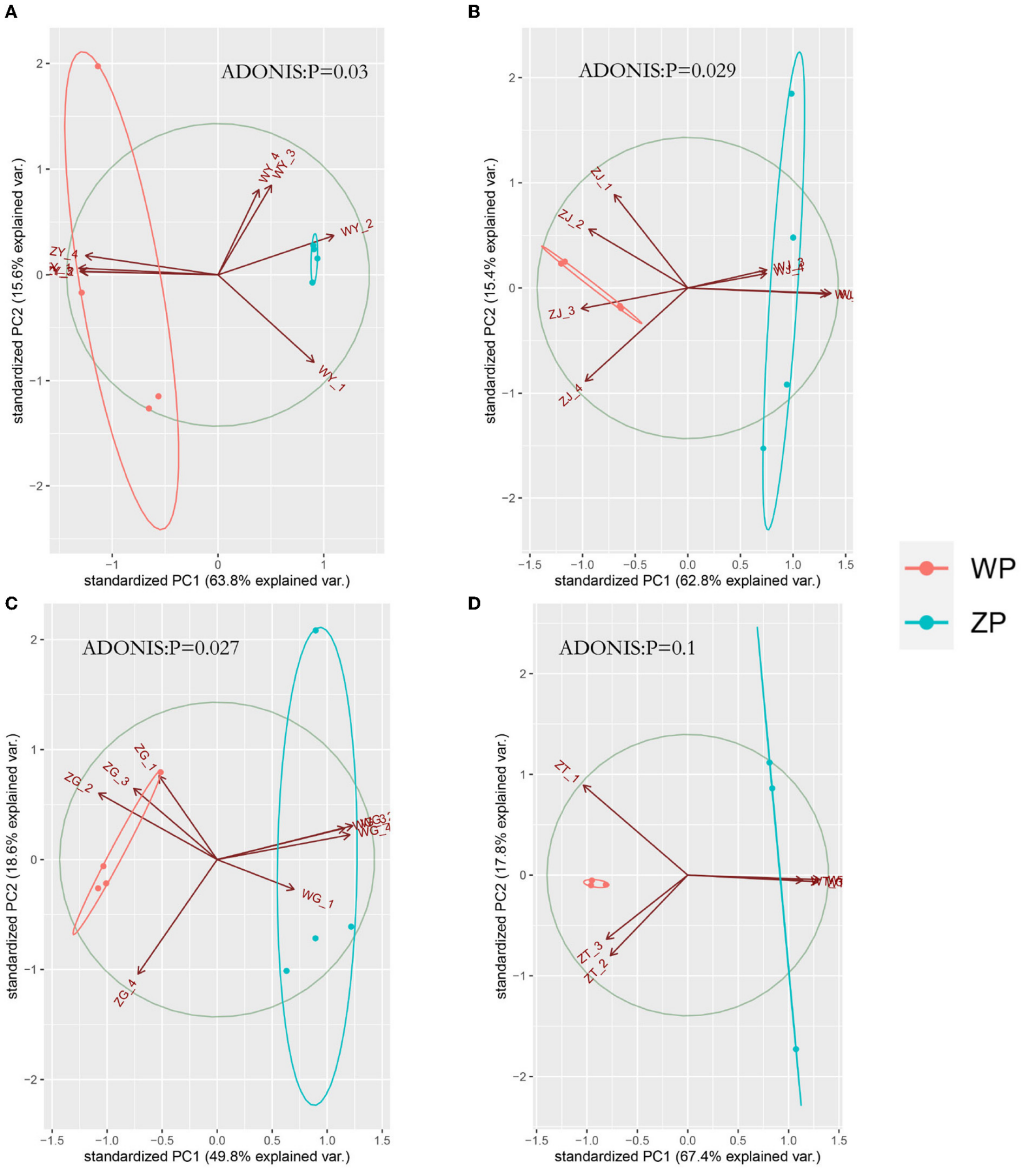
PCA analysis of three compartments between ZP and WP [**(A)** leaf, **(B)** stem, **(C)** root, **(D)** rhizosphere soil].

If we look at the composition of all the communities of the two species, as can be seen from Figure 4A, both ZP and WP are dominated by *Ascomycota*, accounting for an average of 87.83% of the entire community. As the sampling compartment ranges from rhizosphere soil and rhizome to leaf, the relative abundance of Ascomycota in ZP and WP increases in quantity. The Adonis analysis of community composition in different parts of ZP and WP shows significant differences in leaves, stems, and roots (Figure 5).

Furthermore, we performed the ternary phase diagram analysis of the leaves, stems, and roots of two plants (Figure 4B) to determine the distribution of the community in various compartments. We found that a large amount of OUT was shared between stems and leaves. Compared to ZP, the three compartments of WP shared more OTUs, while shared OTUs in ZP tended to occupy a higher proportion of stems and leaves. Based on MetagenomeSeq, the leaves, stems, and roots of ZP and WP were analyzed (Figure 4C). A significant difference in OTUs was found (88) in leaves, 38 in stems, and 71 in roots. We observed the composition of these OTUs at the genus level. In ZP, *Cercospora* (15.34 and 22.11%), *Phaeosphaeria* (8.48, 7.45%), and *Cladosporium* (7.29 and 5.68%) dominate the leaf and stem community, while *Branch06* (30.69%) dominates the root community. In WP, *Cladosporium* (16.02, 20.68, and 8.04%) and *Stachybotryaceae* (17.02, 9.55, and 1.23%) dominate the community of leaves, stems, and roots. In addition, *Plectosphaerella* has a significantly higher abundance in WP than in ZP, especially in stems; 16.29% in WP, compared with only 0.44% in ZP.

### Interaction network analysis of endophytic fungal communities

**Figure 6:**
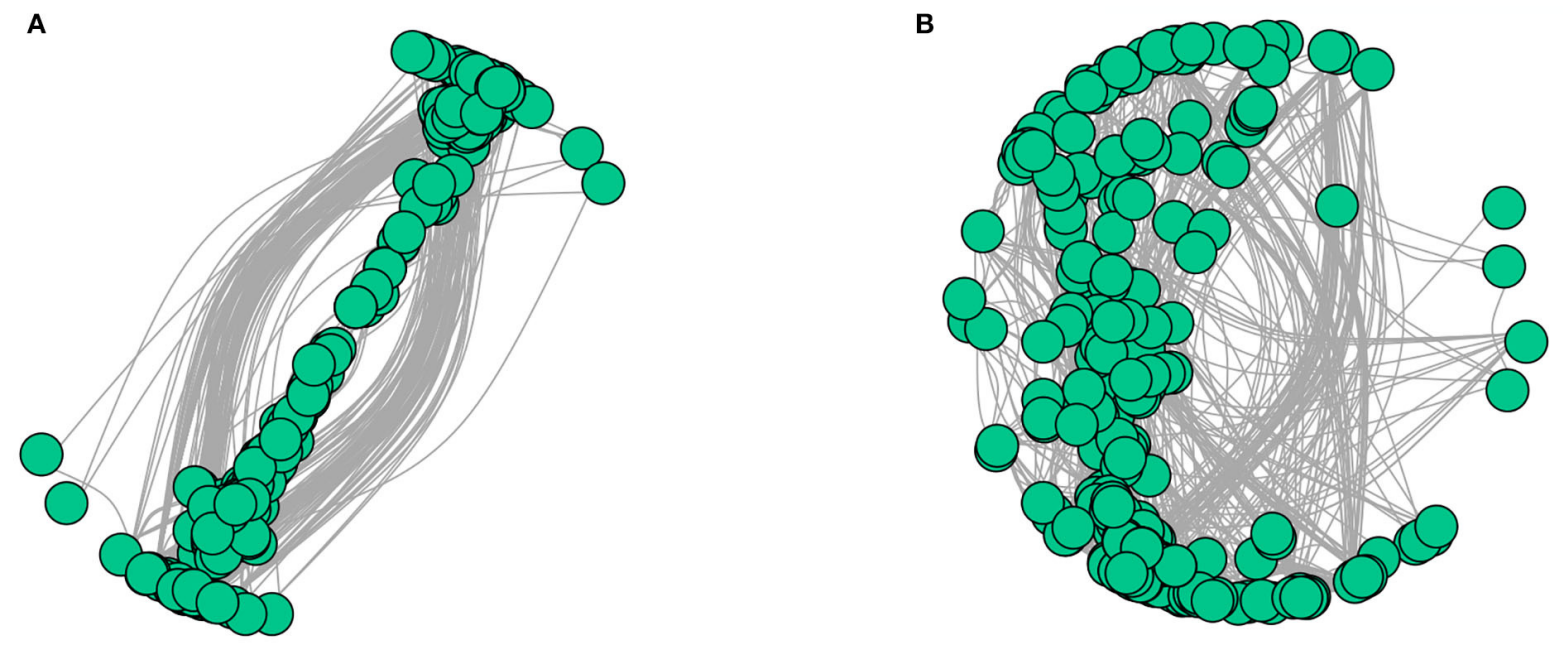
Interaction network diagram of ZP and WP [**(A)** ZP, **(B)** WP].

**Figure 7:**
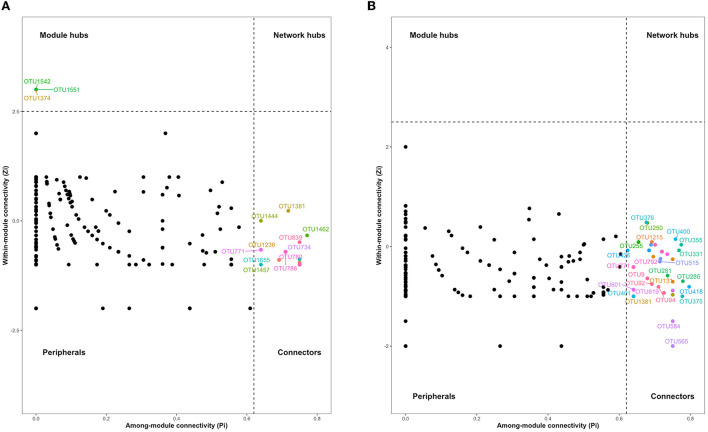
The characteristic annotation analysis of nodes in the interaction network. **(A)** ZP, **(B)** WP.

**Figure 8:**
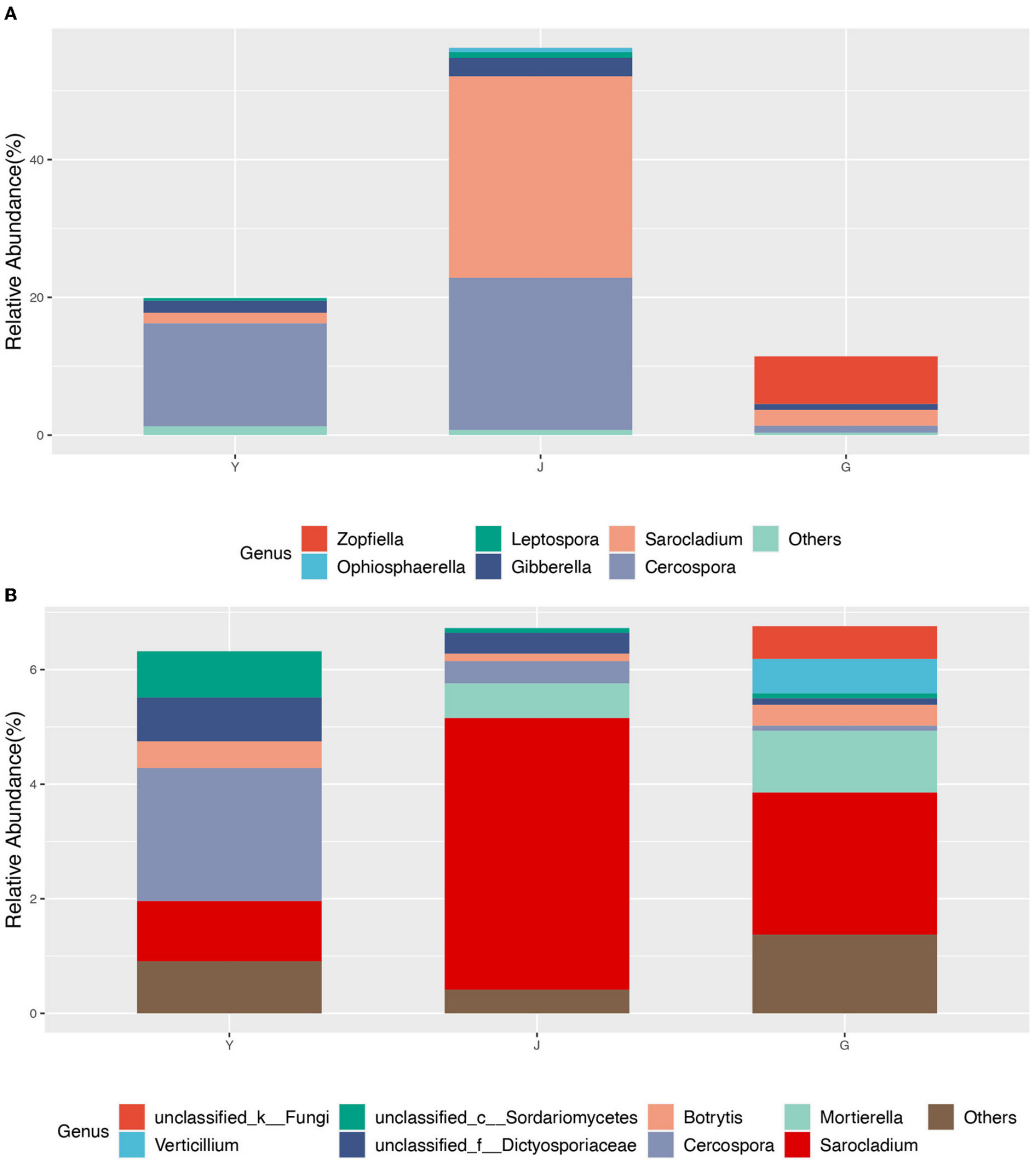
The core community composition at the genus level. **(A)** ZP, **(B)** WP.

The interaction networks of ZP and WP fungal communities were constructed, respectively, and the results are shown in Figure 6. The overall module connectivity of the ZP network is 0.38, and the overall module connectivity of the WP network is 0.34. Still, the nodes of ZP show special aggregation characteristics, while the nodes of WP are more widely distributed, and there is no obvious aggregation. We performed characteristic annotations to the nodes in the network to determine the core community in the endophytic fungal community (Figure 7). This study defines the node annotated as the module node and the connectors as the core community. In ZP, three module hub nodes were identified, including OUT1551 (*Teratosphaeriaceae*), OUT1542 (*Phaeosphaeriaceae*), and OUT1374 (*Basidiomycota*), in addition to 32 connection nodes. In the WP, 74 connection nodes are identified. Observing the community composition at the genus level (Figure 8), the relative abundance of key communities in the three compartments in WP was close, accounting for 19.81% of the total community, dominated by *Sarocladium* in stems and roots and dominated by *Cercospora* in leaves. In ZP, the relative abundance of key communities in the three compartments was significantly different, accounting for 87.59% of the community. The relative abundance of community in stems is highest, dominated mainly by *Sarocladium* and *Cercospora*, in the leaves by *Cercospora*, and in the roots by *Zopfiella*.

### Functional composition analysis of endophytic fungi

**Figure 9:**
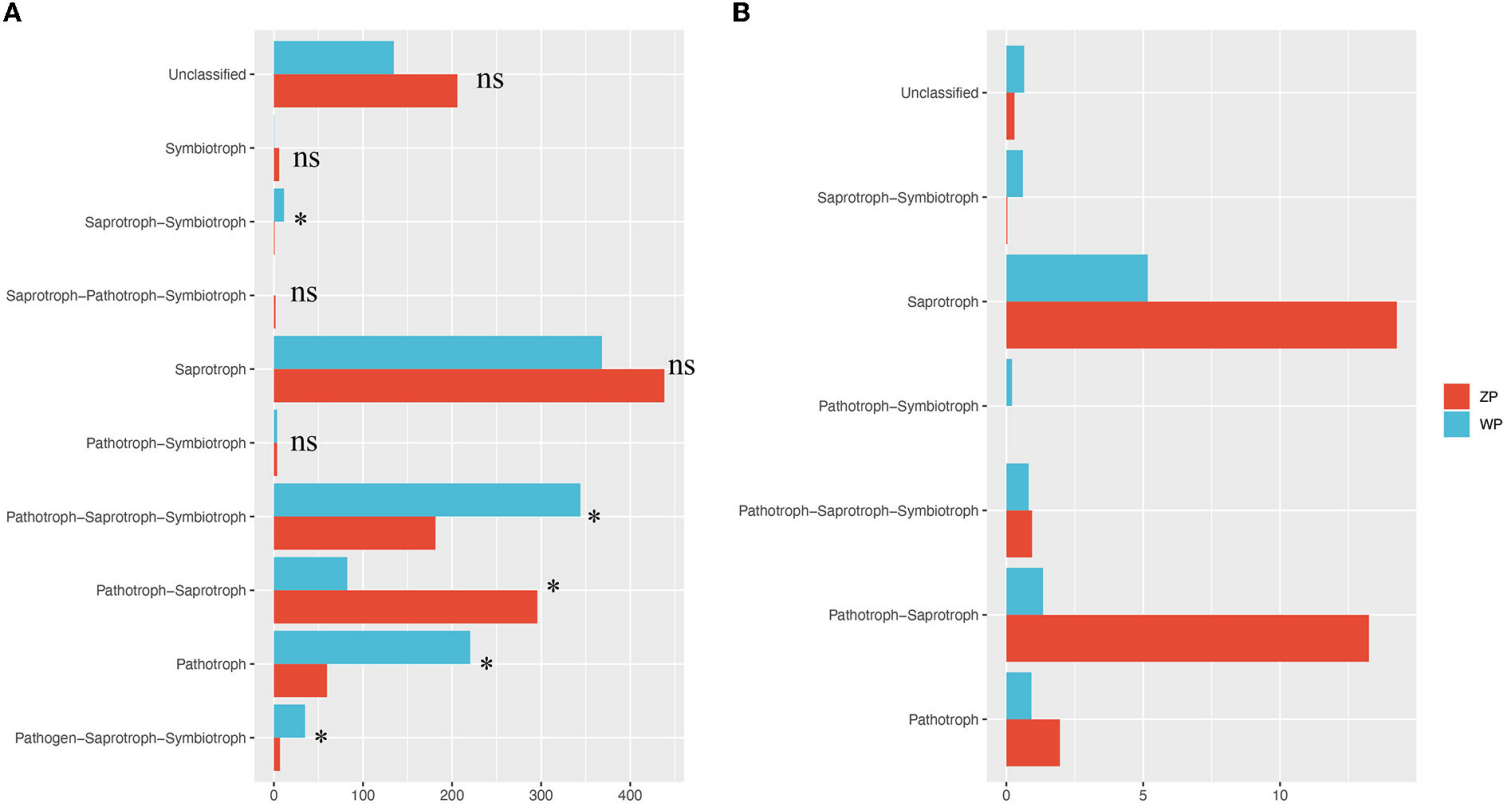
Functional community composition. **(A)** The functional composition of all compartments, **(B)** The functional composition of core community. *P-value < 0.05.

To detect the differences in function community composition in two plants, we used FUNGuild to perform the functional annotations. A total of 10 trophic types are annotated (Figure 9).

**Figure 10:**
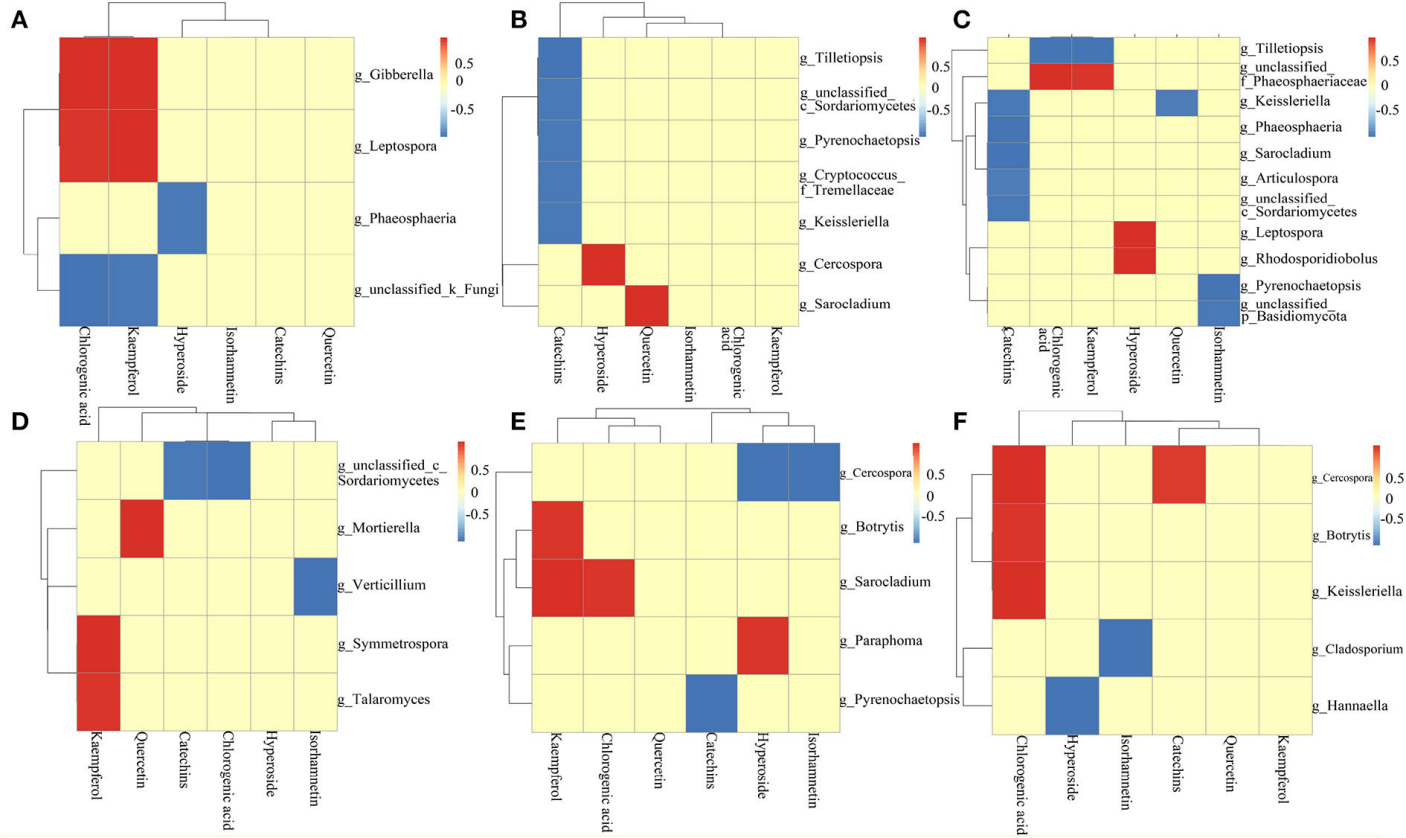
Analysis of the correlation between core community and active ingredient [**(A)** ZP root; **(B)** ZP stem; **(C)** ZP leaf; **(D)** WP root; **(E)** WP stem; **(F)** WP leaf].

We found significant differences in the relative abundance of trophic types between ZP and WP, including pathogen-saprotroph-symbiotroph, pathotroph, pathotroph-saprotroph-saprotroph, pathotroph-saprotroph-symbiotroph, and saprotroph-symbiotroph. Further, a functional analysis of the core community (Figure 10) was performed. In the dominant functional community, saprotrophs and pathotroph-saprotrophs in ZP were significantly higher than in the WP group.

### Correlation analysis of fungal communities with active ingredients

The Pearson-related analysis of core community and active ingredient content in ZP and WP was performed. In the roots of ZP (Figure 10A), *Gibberella* and *Leptospora* were significantly positively correlated with chlorogenic acid and kaempferol. *Phaeosphaeria* was significantly inversely associated with hyperoside. In the stem of ZP (Figure 10B), *Tilletiopsis*, c_*Sordariomycetes, Pyrenochaetopsis*, Cryptococcus_f_*Tremellacea*, and *Keissleriella* were significantly negatively correlated with catechins; *Cercospora* was significantly positively correlated with hyperoside; *Sarocladium* was significantly positively correlated with quercetin. In the leaves of ZP (Figure 10C), *Keissleriella, Phaeosphaeria, Sarocladium, Articulospora*, and c_*Sordariomycetes* were significantly negatively correlated with catechins; *Tilletiopsis* was significantly negatively correlated with chlorogenic acid and kaempferol; f_*Phaeosphaeriaceae* positively correlated with chlorogenic acid and kaempferol; *Leptospora* and *Rhodosporidiobolus* were positively correlated with hyperoside; *Keissleriella* was negatively correlated with quercetin; *Pyrenochaetopsis* and p_*Basidiomycota* were negatively correlated with Isorhamnetin significance.

In the roots of the WP (Figure 10D), *Mortierella, Symmetrospora*, and Taromyces were significantly positively correlated with kaempferol. *Mortierella* was *positively* correlated with quercetin; c_*Sordariomycetes* was significantly inextricably linked to catechins and chlorogenic acids; Verticillium was significantly inversely associated with Isorhamnetin. In the stem of WP (Figure 10E), *Botrytis* and *Sarocladium* were significantly positively correlated with kaempferol; *Sarocladium* was significantly positively correlated with chlorogenic acid; *Paraphoma* was significantly positively correlated with Hyperoside; *Cercospora* was significantly negatively correlated with Hyperoside and Isorhamnetin; *Pyrenochaetopsis* was significantly and negatively correlated with catechins. In the leaves of WP (Figure 10F), *Cercospora, Botrytis*, and *Keissleriella* were significantly positively correlated with chlorogenic acid; *Cercospora* was significantly positively correlated with catechins; *Cladosporium* was significantly negatively correlated with Isorhamnetin; *Hannaella* was significantly negatively correlated with hyperoside.

## Discussion

### The colonization of cercospora interferes with the assembly process of the endophytic fungi of *P. Hydropiper*

In this study, we found a high degree of overlap in the taxa and distribution areas of ZP and WP, and there was no significant difference in the overall alpha diversity between the two. The similarity of fungal communities in rhizosphere soil confirms that both acquire fungi from the same soil species pool. Moreover, the interaction network analysis shows that the connectivity of the endophytic fungi between ZP and WP is very close. These results indicate that the two plants are similar in their ability to shape the composition and structure of endophytic fungal communities. However, we also observed significant leaves, stems, and roots differences between the two plants. This suggests that the assembly process of fungal communities is the key factor influencing the composition of the community. Therefore, we compared the community composition of the three compartments in ZP and WP and assessed the community composition that differed significantly between the two. In ZP, the relative abundance of differential communities in leaves is highest, with *Cercospora* dominating the differential communities in stems and leaves. At the same time, the ACE index of the community in the leaves of ZP was significantly higher than that of WP, indicating a significant enrichment of the community in the leaves of ZP. Studies have shown that *Cercospora* usually colonizes leaves (Khan et al., 2008). Interestingly, the core community in ZP was also dominated by *Cercospora*, accounting for 38.12% of the total community. This suggests that after *Cercospora* colonizes the leaves of ZP, it significantly affects the assembly process of the endophytic fungi. In WP, the relative abundance of differential communities in stems was highest, and the differential community at all three compartments was dominated by *Cladosporium*, which is generally considered to colonize the roots of plants (Hamayun et al., 2009; Chen et al., 2022); this suggests that the assembly process of endophytic fungal communities in WP is still dominated by fungi recruited in the soil.

### Endophytic fungi communitites significantly affect host secondary metabolic processes

We found significantly different communities between ZP and WP, including *Cercospora, Phaeosphaeria, Branch06, Stachybotryaceae*, and *Plectosphaerella*. Most of the community from *Cercospora* is thought to be able to cause diseases in plants. Interestingly, Martínez et al. (2017) found that chlorogenic acid can effectively inhibit the growth of fungi such as *Cercospora*. In this study, the chlorogenic acid content of ZP was significantly higher than that of WP, and the relative abundance of this type of fungi in ZP was also considerably higher than WP, suggesting that it may be due to the extensive colonization of *Cercospora*, resulting in host stress producing more chlorogenic acid. For *Pheosphaeria*, there have been many studies that demonstrate it is rich in secondary metabolites, including polyketone derivatives, pyrazine alkaloids, isocoumarins, perylenequinonoid, anthraquinone, diterpenes, and cyclic peptides (El-Demerdash, 2018; Xiao et al., 2022). This may illustrate the reason that the content of secondary metabolites in ZP is significantly higher than in WP. Fungi, mainly plant pathogens or saprophytic, such as the widely studied *Cladosporium fulvum*, are considered to be the leading cause of moldy tomato leaves. However, many species have also been found in this genus that promotes plant growth, such as through the production of gibberellin (Hamayun et al., 2009, 2010), protein hydrolysates (Răut et al., 2021), and volatile substances (Paul and Park, 2013). In addition, some species from *Cladosporium* can also produce substances such as brefeldin A (Wang et al., 2007) and Cladosins LO (Pan et al., 2020) to play an anti-pathogenic fungus and bacterial activity. It is worth noting that after Ullah et al. (2019) found that the stem of *Poplars* was infected with *Plectosphaerella populi*, the content of catechins and proanthocyanidins increased significantly. In this experiment, the community of *Plectosphaerella* was observed in the stem of WP to be considerably higher than that of ZP. The catechin content of WP was also significantly higher than that of ZP, suggesting that *Plectosphaerella* is likely to be an essential potential community involved in or promoting catechin synthesis and indicating that the synthetic compartment of catechin in the plant may be the stem.

Moreover, we analyzed the functional composition of these communities. In ZP, it is dominated by saprotroph and pathotroph-saprotrophs. In WP, the community of pathotrophs, pathogen-saprotroph-symbiotrophs, and saprotroph-symbiotrophs. Moreover, the relative abundance of the ZP-led functional community was significantly higher than that of WP, and the relative abundance of the dominant community in WP was significantly higher than that of ZP. This indicates a significant difference in the functional community composition between the two. Further, in the functional composition analysis of the core community, it was also found that saprotroph and sathotroph-saprotrophs had significant differences between ZP and WP. These results suggest a difference in the status of endophytic fungi involved in the secondary metabolism of ZP and WP. Further research is needed on the function of these communities in plants.

### The core communities are the essential members involved in the secondary metabolic processes of host plants

We constructed the interaction network of ZP and WP endophytic fungal communities, 3 module hub nodes and 35 connector nodes were found in ZP, and 74 connector nodes were found in WP. The two core communities present different compositions at the genus level, with ZP dominated by Cercospora and Zopfiella. In the WP, it is mainly dominated by Mortierella and *Cercospora*. Kemp et al. (2020) found that *Sarocladium* zeae from this genus acted as systemic endophytic fungi in wheat and as a biological control agent in the host. Since the *Cercospora* and *Sarocladium* play a dominant role in both ZP and WP, therefore, it may indicate that the *Cercospora* has a potential role in regulating the structure of the community. In addition, *Mortierella* is often thought to promote plant growth and increase biomass (Li, S. J. et al., 2018; Ozimek and Hanaka, 2020; Zhang et al., 2020). For *Zopfiella*, Sun et al. (2020) found that species from this genus are capable of producing sesquiterpenes and α-pyranone derivatives.

In the analysis of the correlation between the core community and the active ingredient, 23 distinctly associated communities were found, many of which were thought to promote plant growth and participate in secondary metabolic processes, including *Mortierella* (Li, F. et al., 2018; Ozimek and Hanaka, 2020; Zhang et al., 2020), *Talaromyces* (Naraghi et al., 2012; Lan and Wu, 2020), *Sordariomycete*s (Camarena-Pozos et al., 2021), *Verticillium* (You et al., 2009; Li, N., et al., 2018), *Sarocladium* (Kemp et al., 2020; Błaszczyk et al., 2021; Salvatore et al., 2021), *Gibberella* (Brian et al., 1954; Geng et al., 2014), *Tilletiopsis* (Klecan et al., 1990), and *Phaeosphaeriaceae* (Xiao et al., 2022; Xu et al., 2022), In addition, some plant pathogens are still present in these communities, including *Botrytis, Paraphoma, Cercospora*, and *Pyrenochaetopsis*. These communities potentially affect the secondary metabolic processes of the host, and it is necessary to further identify these communities and their functions in subsequent studies. Therefore, these results demonstrate that the core community of the interaction network plays a vital role in the secondary metabolism of ZP and WP.

## Conclusion

The results showed that the endophytic fungi community assembly processes of *P. hydropiper* and *P. lapathifolium* are significantly different. Since the two acquired fungi from the same soil species pool and have the same ability to shape the composition and structure of endophytic fungal communities, we believe that this difference was mainly due to the infestation of *Cercospora*. In addition, the results also found that the core community was an important member involved in the secondary metabolism of *P. hydropiper* and *P. lapathifolium*. The infection of *Cercospora* provided an opportunity to shape a specific core community. The infection process exerted selective pressure on recruiting other fungi, resulting in a higher proportion of the core community of *P. hydropiper* in the community as a whole than that of *P. lapathifolium*. This increases the odds of the host interacting with the core community. Therefore, it can enhance the communication between host plants and endophytic fungi, affecting the content of secondary metabolites of *P. hydropiper*. Although our study is limited to the two current species, more in-depth studies are needed to elucidate the mechanism by which *Cercospora* infestation affects the process of plant endophytic fungal community assembly. This study has important practical implications for inoculating specific communities in production to improve the yield of crops or medicinal plants. It also provides ideas for the study of the assembly process of other plant endophytic fungi.

## Data availability statement

The data presented in the study are deposited in the NCBI Sequence Read Archive (SRA) database repository, https://www.ncbi.nlm.nih.gov/, accession number SRR20897189-SRR20897218.

## Author contributions

XZ, HL, QH, and JW conceived and designed the study. XZ, HL, MT, ZD, and QF performed the experiments. XZ and HL processed the data and wrote the article. All authors read and approved the manuscript.

## Funding

This work was financially supported by the Sichuan Science and Technology Department (2021ZYD0109 and 2022YFS0430). We are indebted to our alma mater, Chengdu University of Traditional Chinese Medicine, for the convenience of collecting documents and experiments. Thanks for all the help from everyone in our lab.

## Conflict of interest

Author JS was employed by Sichuan Fuzheng Pharmaceutical Co., Ltd.

The remaining authors declare that the research was conducted in the absence of any commercial or financial relationships that could be construed as a potential conflict of interest.

## Publisher's note

All claims expressed in this article are solely those of the authors and do not necessarily represent those of their affiliated organizations, or those of the publisher, the editors and the reviewers. Any product that may be evaluated in this article, or claim that may be made by its manufacturer, is not guaranteed or endorsed by the publisher.
