# Supplementary material for: Colonization characteristics of fungi in Polygonum hydropipe L. and Polygonum lapathifolium L. and its effect on the content of active ingredients
Source: Front Plant Sci. 2022 Sep 28;13:984483. doi: 10.3389/fpls.2022.984483 (PMC9554492; doi:10.3389/fpls.2022.984483)
Supplement: Supplementary file 1 [file Table_1.DOCX]

Supplemental table 1 Optimized MS /MS parameters of ZP

| Compound | Molecular formula | MRM parameters | | | | | |
| --- | --- | --- | --- | --- | --- | --- | --- |
|  |  | Mass data( m/z) | DP /V | | CE/eV | CE/eV  CXP /eV | Ion mode |
| Catechins | C_15_H_14_O_6_ | 291.10/139.0 | 90 | 14 | | 14 | ESI+ |
| Chlorogenic acid | C_16_H_18_O_9_ | 353.1 /190.9 | -35 | -20 | | -14 | ESI- |
| Hyperoside | C_21_H_20_O_12_ | 462.9/300.0 | -155 | -36 | | -14 | ESI- |
| Quercetin | C_21_H_20_O_11_ | 301.1/151.0 | -155 | -14 | | -14 | ESI- |
| Kaempferol | C_15_H_10_O_6_ | 285.0 /116.9 | -120 | -36 | | -14 | ESI- |
| Isorhamnetin | C_16_H_12_O_7_ | 314.9 /300.0 | -120 | -25 | | -14 | ESI- |
